# Control of Dopamine Signal in High-Order Receptor Complex on Striatal Astrocytes

**DOI:** 10.3390/ijms25168610

**Published:** 2024-08-07

**Authors:** Sarah Amato, Monica Averna, Elisa Farsetti, Diego Guidolin, Marco Pedrazzi, Elena Gatta, Simona Candiani, Guido Maura, Luigi Francesco Agnati, Chiara Cervetto, Manuela Marcoli

**Affiliations:** 1 Department of Pharmacy, Section of Pharmacology and Toxicology, University of Genova, Viale Cembrano 4, 16148 Genova, Italy; 2 Department of Experimental Medicine, Section of Biochemistry, University of Genova, Viale Benedetto XV 1, 16132 Genova, Italy; 3 Department of Neuroscience, University of Padova, Via Gabelli 63, 35122 Padova, Italy; 4 DIFILAB, Department of Physics, University of Genova, Via Dodecaneso 33, 16146 Genova, Italy; 5 Department of Earth, Environment and Life Sciences, University of Genova, Viale Benedetto XV 5, 16132 Genova, Italy; 6 IRCCS Ospedale Policlinico San Martino, Via Largo Benzi 10, 16132 Genova, Italy; 7 Department of Biomedical, Metabolic Sciences and Neuroscience, University of Modena and Reggio Emilia, 41121 Modena, Italy; 8 Interuniversity Center for the Promotion of the 3Rs Principles in Teaching and Research (Centro 3R), 56122 Pisa, Italy

**Keywords:** receptor mosaic, high-order receptor complex, heteromers, astrocyte process, glutamate, intracellular calcium, neuroglia, oxytocin, adenosine receptor, dopamine receptor

## Abstract

The receptor–receptor interaction (RRI) of G protein-coupled receptors (GPCRs) leads to new functional entities that are conceptually distinct from the simple addition of signals mediated by the activation of the receptors that form the heteromers. Focusing on astrocytes, there is evidence for the existence of inhibitory and facilitatory RRIs, including the heteromers formed by the adenosine A2A and the dopamine D2 receptors, by A2A and the oxytocin receptor (OTR), and the D2-OTR heteromers. The possible involvement of these receptors in mosaicism has never been investigated in striatal astrocytes. By biophysical and functional approaches, we focused our attention on the existence of an A2A-D2-OTR high-order receptor complex and its role in modulating cytosolic calcium levels and endogenous glutamate release, when striatal astrocyte processes were stimulated with 4-aminopyridine. Functional data indicate a permissive role of OTR on dopamine signaling in the regulation of the glutamatergic transmission, and an inhibitory control mediated by A2A on both the D2-mediated signaling and on the OTR-facilitating effect on D2. Imaging biochemical and bioinformatic evidence confirmed the existence of the A2A-D2-OTR complex and its ternary structure in the membrane. In conclusion, the D2 receptor appears to be a hotspot in the control of the glutamate release from the astrocytic processes and may contribute to the regulation and integration of different neurotransmitter-mediated signaling in the striatum by the A2A-D2-OTR heterotrimers. Considering the possible selectivity of allosteric interventions on GPCRs organized as receptor mosaics, A2A-D2-OTR heterotrimers may offer selective pharmacological targets in neuropsychiatric disorders and neurodegenerative diseases.

## 1. Introduction

G protein-coupled receptors (GPCRs) are the major druggable targets expressed on the cell membrane. Their structure is characterized by seven transmembrane domains with an extracellular N-terminus and a cytosolic C-terminus. Endogenous substances activating GPCRs include amino acids, peptides, proteins, lipids, purine nucleosides, and nucleotides. The activation of GPCRs leads to signal transduction through heterotrimeric G-proteins that regulate all kinds of cellular events. GPCRs can work as homomeric functional units [1,2] or as heteromers [3,4,5,6,7,8,9,10]. In recent years, the existence of GPCR heteromers and their role in the molecular architecture of the central nervous system (CNS; [11]) and in the neuron–astrocyte cross talk have been widely accepted [12,13]. The heteromerization of GPCRs results in new functional entities, conceptually different from the simple addition of signals mediated by the activation of the receptors that make up the heteromer. The architecture of GPCR homomers or heteromers is due to direct physical contact between monomers of the same type, monomers for the same transmitter (e.g., for the adenosine receptors A2A-A1 or the metabotropic glutamatergic receptors mGluR3-mGlur5), or for different chemical signals (e.g., for the adenosine receptor A2A and the dopamine receptor D2 in A2A-D2 heteromers; [14,15,16]). The resulting novel entity acquires a different biochemical profile based on reciprocal allosteric modulations. These new receptor assemblies generated by direct receptor–receptor interaction (RRI) could be considered as a sophisticated and flexible cell-decoding apparatus. In fact, due to reciprocal allosteric modulation, GPCR complexes operate as highly plastic integrative units [15,17,18,19,20,21,22,23]. The RRI is an efficient and adaptable way to modulate the properties of the receptors involved, offering a new model for the regulation and integration of extracellular signaling [19,20]. In neuroscience, many efforts have been made to elucidate the mechanisms of signaling integration at the heteromers in intercellular communication. Research focusing on astrocytes, one of the most numerous cell types in the CNS, has provided substantial evidence for the presence of RRIs ([24] and references inside). Notable examples include the putative RRI between the metabotropic glutamatergic receptor mGluR3-mGluR5 [25], the probable heteromers between the GABAB receptor and the somatostatin SSTR4 [26], or the known heteromers involving adenosine A2A or dopamine D2 receptors, such as D2-5HT1 [27]. In particular, we have reported that the heteromers formed by the adenosine A2A and the dopamine D2 receptors (A2A-D2) [28,29,30], by the adenosine A2A and the oxytocin receptor OTR (A2A-OTR) [31], and by the dopamine D2 receptor and OTR (D2-OTR) [32] in striatal astrocytic processes could modulate the efflux of glutamate evoked by a physiological depolarization. Here, we focus on whether a high-order receptor complex A2A-D2-OTR can also be present in adult astrocytes, and, in particular, on the astrocytic processes. We assessed the possible functional modulation of the intracellular [Ca^2+^] and the release of the gliotransmitter glutamate from striatal astrocytic processes. Moreover, we used a molecular modeling study to predict possible interaction interfaces between the receptors.

## 2. Results

### 2.1. On Striatal Astrocytes the OTR, A2A, and D2 Receptors Are Co-Localized on GFAP–Ezrin Positive Processes

**Figure 1:**
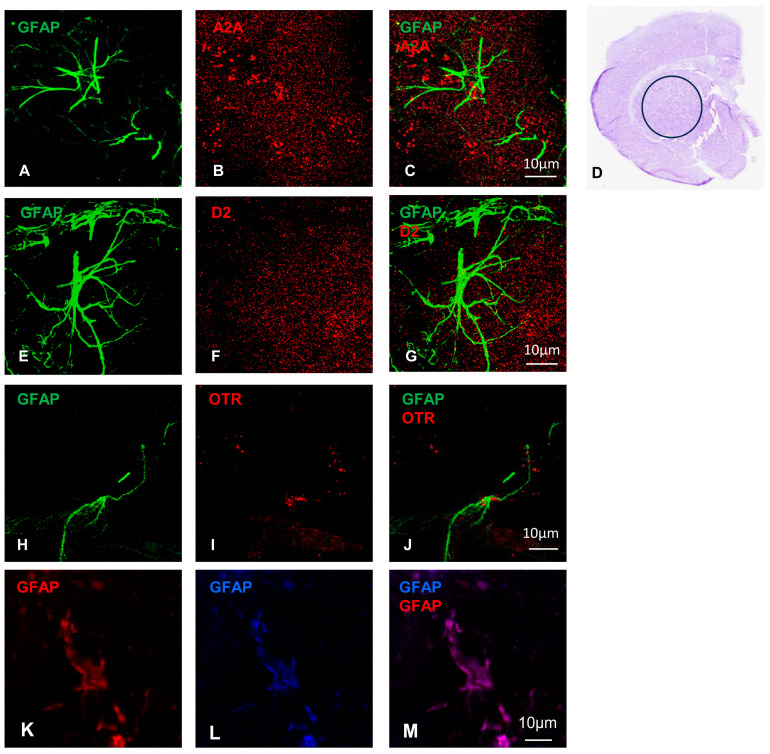
Striatal astrocytes express the A2A, D2, and OT receptors. Representative confocal images showing the presence of the A2A, D2, and OTR in rat striatum and their co-localization with GFAP. Immunofluorescence analysis was conducted in rat hemibrain slices (see Section 4 for details). Maximum intensity projections and their merges for representative fields (with a dimension of 60 × 60 µm; at least 11 z-stacks) are shown; GFAP (green, (**A**,**C**,**E**,**G**,**H**,**J**)), A2A (red, (**B**,**C**)), D2 (red, (**F**,**G**)), and OTR (red, (**I**,**J**)). Astrocytes were positively stained with mouse anti-GFAP (red (**K**,**M**)) and goat anti-GFAP (blue (**L**,**M**)) primary antibodies. The scale bars are indicated in the merged images. (**D**) Cresyl violet staining of a close-up section used in Figure 2. GFAP, glial fibrillary acidic protein; A2A, adenosine receptor; D2, dopamine receptor; OTR, oxytocin receptor.

In the striatum, the presence of A2A, D2, and OT receptors on astrocytes was examined by immunofluorescence. Slices of rat hemibrain were incubated with the specific astrocytic markers GFAP, and the astrocytes resulted as GFAP-positive cells (Figure 1). Using anti-A2A, anti-D2, or anti-OTR primary antibodies, we observed a localization of the receptors on GFAP (Figure 1). These data are consistent with previous observations in the adult rat striatum [28,29,31,32] and indicate that GFAP-positive astrocytes express OTR, A2A, and D2 receptors.

**Figure 2:**
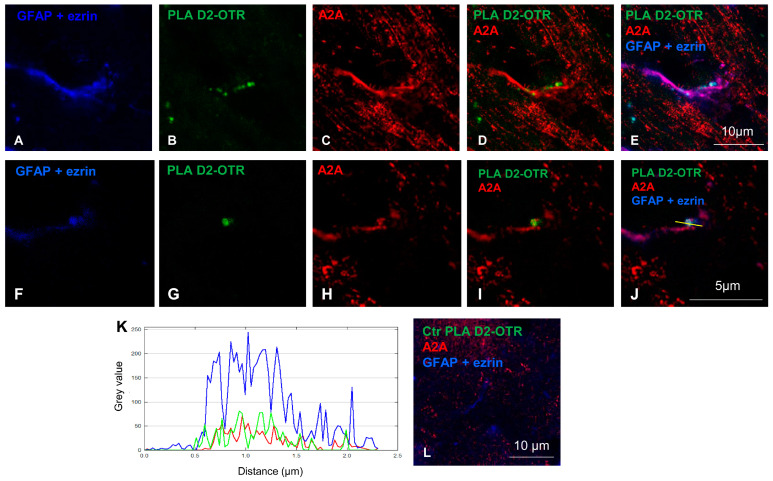
Astrocytes express the A2A receptors and D2-OT heteromers. Representative confocal images showing the presence of the D2-OTR heteromers in rat striatum and their co-localization with A2A receptors. The immunofluorescence analysis was conducted by combing the in situ PLA with anti-D2 receptor and anti-OTR primary antibodies, and the classical immunofluorescence technique using anti-A2A receptor, anti-GFAP, and anti-ezrin primary antibodies in rat hemibrain slices (for further details, see Materials and Methods). (**A**–**E**) Maximum intensity projections for single channels and the merged images of a representative field (29.13 × 29.13 µm; 11 z stacks) are shown; GFAP + ezrin (blue, (**A**,**E**)), PLA D2-OTR (green, (**B**,**D**,**E**)), and A2A (red, (**C**–**E**)). (**F**–**J**) Enlarged images for each channel and the merged confocal images of a single z-stack is shown; this is the same field of the maximum intensity projections shown in (**A**–**E**); GFAP + ezrin (blue, (**F**,**J**)), PLA D2-OTR (green, (**G**,**I**,**J**)), and A2A (red, (**H**–**J**)). The yellow line in (**J**) was used to create the profile plot shown in (**K**). The colours of the different lines (blue for the GFAP + ezrin signal, green for the PLAD2-OTR signal, red for the A2A signal) correspond to those used in image (**J**). (**L**) A representative merge control image obtained without the primary antibody for OTR or D2 during the PLA protocol. GFAP, glial fibrillary acidic protein; A2A, adenosine receptor; D2, dopamine receptor; OTR, oxytocin receptor; PLA, proximity ligation assay.

Using the proximity ligation assay (PLA), we previously demonstrated the presence of heteromers A2A-D2, A2A-OTR, and D2-OTR in the dorsal and ventral striatal astrocytes on rodent slices. Here, we investigated the colocalization of the D2-OTR heteromers with the A2A receptors on astrocytes positive for the glial markers glial fibrillary acidic protein (GFAP) and ezrin. In situ PLA allows one to identify the physical closeness of proteins, and the corresponding signal is only produced if the proteins are strictly close (~16 nm; [33]). Here, the PLA revealed D2-OTR heterodimer complexes as spots in GFAP and ezrin-positive astrocytes, as shown in a maximum intensity projection (Figure 2). The overlapping image of PLA D2-OTR and A2A receptors shows the co-expression of D2-OTR with the A2A receptors as yellow pixels (Figure 2D,I), or as white pixels (Figure 2E,J) when all the receptors are co-localized on GFAP and ezrin-positive structures. The co-localization is shown in a single z-stack (Figure 2I,J), while the single labelings are shown in Figure 2F–H. On the enlarged triple labeling image (Figure 2J), a profile plot (Figure 2K) was generated along the yellow line with a length of 2.25 µm (Figure 2J). As a negative control, in some experiments, we omitted one of the two primary antibodies required for the ligation assay, and the PLA signal was not detected (Figure 2L).

This finding indicates that the striatal astrocytes are endowed with all three GPCRs, A2A, D2, and OT receptors, and with A2A receptors co-localized with D2-OTR heteromers, possibly allowing an A2A-D2-OTR mosaic. 

### 2.2. On Striatal Astrocytic Processes the A2A, D2 and OT Receptors Are Co-Localized

**Figure 3:**
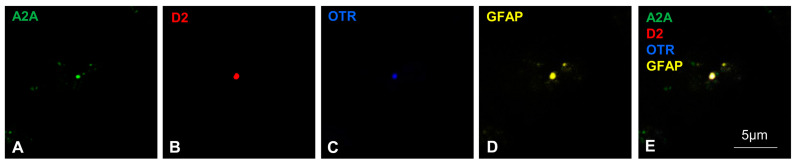
Astrocytic processes express A2A, D2, and OTRs. Representative confocal images showing the presence of the studied receptors in rat striatal astrocytic processes and their co-localization with GFAP. The immunofluorescence analysis was conducted using anti-D2 receptor, anti-OTR, anti-A2A receptor, and anti-GFAP primary antibodies in gliosomes (for further details, see Materials and Methods). (**A**–**E**) A representative field is shown; A2A (green, (**A**,**E**)), D2 (red, (**B**,**E**)), OTR (blue, (**C**,**E**)), and GFAP (yellow, (**D**,**E**)). GFAP, glial fibrillary acidic protein; A2A, adenosine A2A receptor; D2, dopamine D2 receptor; OTR, oxytocin receptor.

The gliosomes were examined for the triple co-localization of the receptors studied. The fine perisynaptic astrocytic processes (PAPs) were previously characterized [29] using specific astrocytic markers such as GFAP and ezrin [34] and were positively labeled with the vesicular glutamate transporter type 1, VGLUT 1 [29]. We have demonstrated that striatal gliosomes were endowed with the A2A, D2, and OT receptors [28,29,31,32]. Here, we labeled the astrocytic particles with anti-OTR, anti-D2, and anti-A2A primary antibodies, and obtained evidence of the co-expression of the three receptors on the same gliosomes (Figure 3A–E). This finding indicates that the same astrocyte processes are endowed with all three GPCRs, A2A, D2, and OTR, possibly allowing an A2A-D2-OTR mosaic in striatal astrocytes.

### 2.3. On the Membrane of Striatal Astrocytic Processes, the A2A, OTR and D2 Receptors Physically Interact

**Figure 4:**
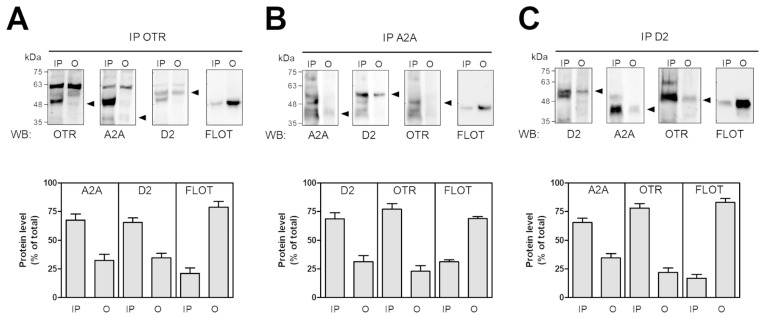
Co-immunoprecipitation of the OTR, A2A, and D2 receptors in striatal astrocytic processes. The aliquots (300 µg) of Triton X-100-soluble proteins prepared from striatal fresh isolated gliosomes were immunoprecipitated with 1 µg of anti-OTR (**A**), or anti-A2A (**B**), or anti-D2 (**C**) antibody. The immunoprecipitated (IP) and not immunoprecipitated (O, output) materials were analyzed by Western blot (WB) for the indicated antigens. A2A, OTR, D2, and flotillin-1 (FLOT) immunoreactive bands were quantified and the data are reported in the graphs as a percentage of the total amount of the relevant protein (% of total). The values are means ± SEM (*n* = 5 for (**A**,**C**); *n* = 4 for (**B**)). For each protein, a representative blot is shown. The arrows indicate the expected weights of the antigens. A2A, adenosine A2A receptor; D2, dopamine D2 receptor; OTR, oxytocin receptor; FLOT, flotillin.

To assess the ability of physical interactions between the studied GPCRs on astrocytes, we performed an immunoprecipitation assay on the membrane of the freshly prepared striatal gliosomes. By co-immunoprecipitation, we found that the OTR physically interacts with the A2A and D2 receptors (Figure 4A); similar interactions were found when we used the anti-A2A or anti-D2 receptor antibodies (Figure 4B,C). In all the experimental immunoprecipitation conditions, the co-immunoprecipitation of flotillin-1, a marker of membrane lipid rafts (Figure 4A–C), suggests that the three GPCRs are expressed in the lipid domain.

### 2.4. Functional Evidence for a Putative A2A-D2-OTR Mosaic: D2-OTR Heteromer-Mediated Inhibition of the 4-AP-Evoked Calcium Signals Is Nullified by A2A Receptor Activation

**Figure 5:**
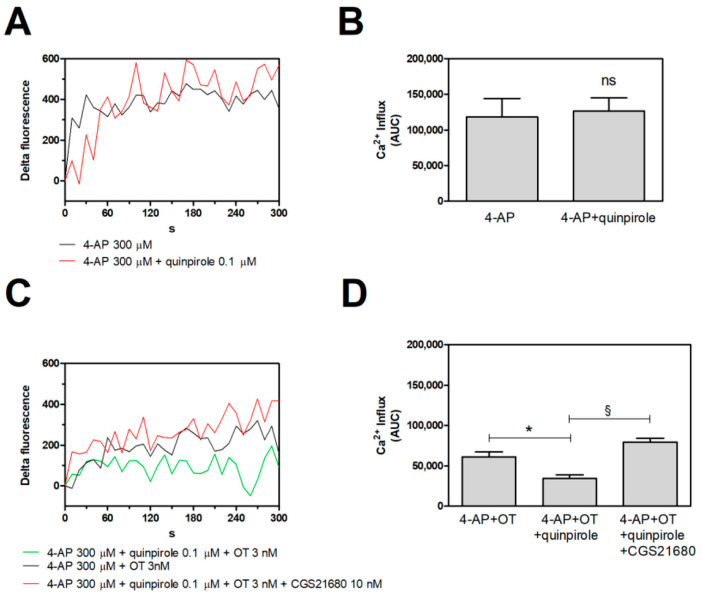
CG-loaded gliosomes were exposed to the indicated stimuli for 300 s at 37 °C. (**A**–**C**), CG-dependent fluorescence was monitored every 10 s from 0 to 300 s. [Ca^2+^]_i_ increase is expressed as “Delta Fluorescence”. The lines represent the mean values from 7 to 3 experiments (**A**) and from 5 independent experiments (**C**). (**B**–**D**), the Ca^2+^ influx after 300 s was estimated by calculating the areas underlying the curves (AUC) and is reported in the graphs for each experimental condition. Data are means ± SEM. ns, not statistically significant according to the Mann–Whitney test. * *p* < 0.01 and § *p* < 0.001 according to ANOVA, followed by Tukey’s post hoc test. 4-AP, 4-aminopyridine; CG, Calcium Green™-1 AM; OT, oxytocin.

As previously described by Amato and co-workers [32], the activation of OTR and D2 receptors induces a significant reduction in the endogenous glutamate release evoked by 4-aminopyridine (4-AP). In this study, we evaluated their functional interaction on the Ca^2+^ signals and explored the potential allosteric control mediated by the A2A receptor activation. The D2 agonist quinpirole (0.1 µM) per se was unable to affect the intracellular Ca^2+^ levels evoked by 4-AP in striatal processes (Figure 5A,B). We previously demonstrated that the activation of the OTR by oxytocin (OT) 3 nM significantly reduced the 4-AP-evoked Ca^2+^ signals in the processes [31]. As observed for glutamate release [32], the co-activation of the OTR and of the D2 receptor by quinpirole 0.1 µM determined a robust inhibition of the 4-AP-evoked Ca^2+^ signals in the processes when the OTR is activated by OT 3 nM (Figure 5C,D), further demonstrating a RRI through which the OTR and D2 receptors regulate the intracellular [Ca^2+^] in the striatal network. The presence of the A2A receptor agonist, CGS 2718 (0.01 µM), abolished the inhibition exerted by the OTR activation and its facilitating effect on the D2 receptors in the control of the depolarization-evoked Ca^2+^ signals (Figure 5C,D).

### 2.5. Functional Evidence for a Putative A2A-D2-OTR Mosaic: D2-OTR Heteromer-Mediated Inhibition of the 4-AP-Evoked Glutamate Release Is Nullified by A2A Receptor Activation

Previously, we demonstrated the presence of heteromers A2A-D2 [28,29,30], A2A-OTR [31], and D2-OTR [32], with functional implications in the control of the 4-AP-evoked glutamate release and calcium signals. As assessed by confocal microscopy, the A2A, D2, and OT receptors were expressed in the same astrocyte processes. Here, we evaluated the possible functional interaction with all three receptors in a mosaic structure and its ability to modulate glutamate release evoked by a similar physiological stimulus during superfusion.

**Figure 6:**
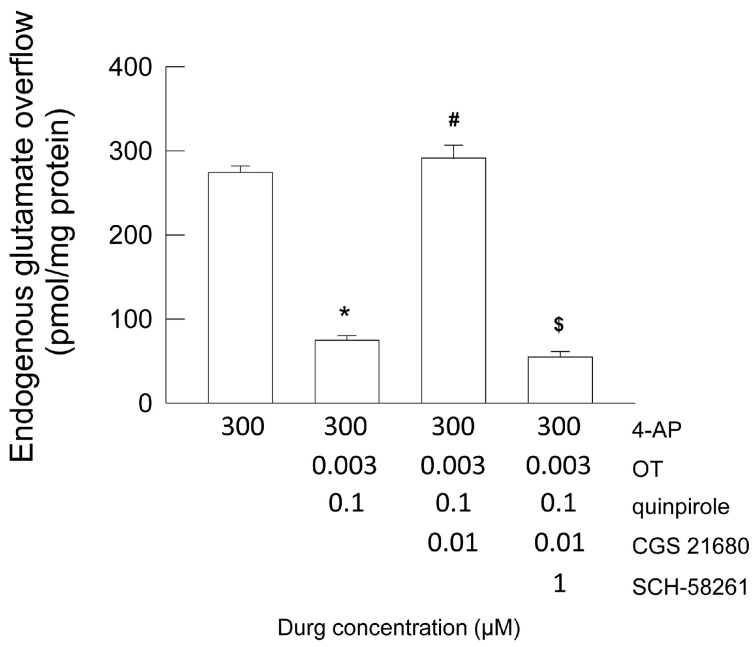
Endogenous glutamate efflux in response to 4-AP-induced depolarization in striatal gliosomes. Modulation by the D2-OTR heteromers and A2A receptors. The inhibitory effect of OT 3 nM and quinpirole 0.1 µM on the 4-AP-evoked endogenous glutamate release; abolishment by CGS 21,680 0.01 µM and the antagonism of the A2A receptors by SCH-58261. The bars represent the overflow of the glutamate release, expressed as pmol/mg of protein, in the presence of drugs at the concentrations used. Briefly, 4-AP was added (6 min) during superfusion; OT, quinpirole, and CGS 21,680 were added together with 4-AP, while SCH-58261 was added 8 min before the agonists. Further experimental details can be found in Materials and Methods. Data are the mean ± SEM of *n* = 5–10 independent experiments. * *p* < 0.0001 compared with the effect of 4-AP; # *p* < 0.0001 compared with the effect of 4-AP + OT + quinpirole; $ *p* < 0.0001 compared with the effect of 4P + OT + quinpirole + CGS 21,680 by one-way ANOVA plus Tukey’s post hoc test. 4-AP, 4-aminopyridine; OT, oxytocin.

In the first two fractions collected, superfusion with the physiological standard medium of striatal gliosomes caused a glutamate efflux equivalent to 82.23 ± 4.20 pmol/mg protein min (n = 10). The stimulation with 4-AP (300 µM) increased the efflux, and the evoked overflow was 274.30 ± 7.85 pmol/mg protein; n = 10 (Figure 6). The co-activation of OTR by OT 3 nM and of the D2 receptor by quinpirole 0.1 µM determined a robust inhibition of the 4-AP-evoked glutamate release, indicating a strictly functional collaboration of the OTR and D2 receptors in the control of the gliotransmitter efflux. We have to remember that the D2 agonist, quinpirole 0.1 µM, was ineffective in the modulation of the endogenous glutamate release in basal condition as in the presence of 4-AP, and that OT 3 nM had a facilitatory effect on the D2 receptor response through a facilitatory D2-OTR RRI [32]. Moreover, the presence of the A2A receptor agonist, CGS 2718 (0.01 µM), nullified the inhibition exerted by OTR activation and its facilitation effect on D2 receptors in the control of glutamate release (Figure 6). The A2A antagonist, SCH-58261, annulled the A2A effect (Figure 6). Collectively, the findings indicate that activation of A2A controlled the OTR-D2 allosteric interaction in the inhibition of the glutamate release in striatal astrocyte processes.

### 2.6. Estimated Model of a Putative A2A-D2-OTR Mosaic

**Figure 7:**
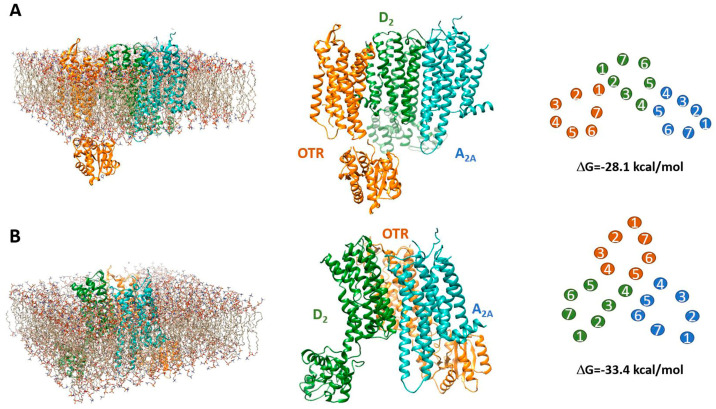
Predicted models of the A2A-D2-OTR heterotrimer. The predicted structure of the receptor complex in an “open” topological arrangement is shown in (**A**). It was derived by first docking A2A and D2 receptors and then associating the OTR. The left panel shows the complex in the membrane environment used for refinement by energy minimization and the resulting heterotrimer is shown in the middle panel. A solution characterized by a “closed” topological arrangement is shown in (**B**). It was obtained by docking the A2A receptor to the previously estimated D2-OTR heterodimer. For both cases, the obtained arrangement of the transmembrane domains (as different coloured numbers for each receptor in the illustration) is illustrated in the right panels together with the estimated change in free energy associated with complex formation. For each topology, the structure shown is the one exhibiting the best energy score according to the applied docking method.

The 50 best solutions estimated by the applied docking protocol (and following refinement by energy minimization) suggest that the adenosine A2A, dopamine D2, and oxytocin receptors might form receptor mosaics according to two topologies. In about 80% of the obtained solutions, indeed, heterotrimers followed an “open” (linear) geometry in which the receptor complex was characterized by two interfaces, allowing the aggregation of two protomers to the third one. A “closed” (triangular) topology was exhibited by the remaining 20% of the obtained solutions, in which three interfaces were created in the heterotrimer by the interaction of each protomer with both partners. Two examples are provided in Figure 7. They represent the estimated solutions with the best energy score in the two cases.

## 3. Discussion

The collected evidence indicates the existence of a functional A2A-D2-OTR mosaic on striatal astrocytes: (1) the A2A receptors colocalized with D2-OTR heteromers in native striatal astrocytes; (2) in striatal perisynaptic astrocyte processes, the A2A, D2, and OT receptors were colocalized on membranes; (3) they were co-immunoprecipitated in protein complexes in which flotillin-1 was present; (4) the A2A receptor activation abolished the OTR inhibition of the glutamate efflux and nullified its permissive role on the D2 receptor control of the gliotransmitter release and of the intracellular calcium modulation; (5) a bioinformatic study predicted that linear or triangular geometries may characterize the structures of the A2A-D2-OTR heterotrimer.

The reported biophysical, biochemical, and functional evidence was collected in slices and in freshly isolated astrocyte processes prepared from adult rat striatum. Striatal gliosomes were obtained from the branches of the astrocytes maturated in the in vivo network, where astrocytes and neurons communicate to maintain the homeostasis of the striatal functions. In the mammalian brain, astrocytes, one of the most numerous cell types in the CNS, play roles in high-level brain integrative functions [35,36], and are involved in the fine regulation of the glutamatergic synaptic transmission [37,38,39]. At the tripartite synapses, astrocytic cells can interact with neurons at a functional level and modulate synaptic transmission and neuronal plasticity [37,40,41]. In fact, the astrocyte–neuron interaction occurs since astrocytic cells express GPCRs and ligand-gated ion channels for many neurotransmitters (see [42,43,44]) and are able to release gliotransmitters, whose receptors are located at pre- or postsynaptic neurons [45]. Considering the glutamatergic transmission, astrocytes also express glutamatergic transmembrane transporters that efficiently remove glutamate from the synaptic cleft, controlling its concentration and, consequently, the excitatory transmission [46,47]. Perivascular astrocyte end-feet surround the brain capillaries, placing astrocytes in a strategic position between capillaries and neurons [48] and in the control of the small molecules and ions flow through their end-feet processes at the blood–brain barrier [49,50].

PAPs are integral functional elements of tripartite glutamatergic synaptic complexes [37], with key roles at glutamatergic synapses. In neuron–astrocyte networks, PAPs actively modulate neuronal activity by regulating extracellular space volume and synapse coverage [51] through glutamate clearance and by releasing gliotransmitters (see [52,53,54,55,56]).

The relevance of the function of astrocytes and of the neuron–astrocyte network in vulnerability to neurodegenerative and neuropsychiatric diseases is long recognized [36,57,58,59]. Astrocyte dysfunction at tripartite synapses and altered striatal glutamatergic neurotransmission have emerged in schizophrenia [60,61,62]. In the striatum, astrocytes may be directly involved in behavioral aspects such as emotion, cognition, and sensory processing [63] as in substance abuse disorders [64]. There is also increasing evidence that astrocytes play a role in Parkinson’s disease (PD), with functional plastic changes at striatal glutamatergic synapses, dysregulated biology, and the upregulation of gene expression. In the PD model of the 1-methyl-4-phenyl-1,2,3,6-tetrahydropyridine (MPTP)-treated monkeys, a remodeling of the pre- and postsynaptic neuronal elements at the cortico-striatal and thalamo-striatal axo-spinous synapses has been observed [65,66,67], and it has been reported that in PD at the same synapse perisynaptic astrocytes undergo a significant ultrastructural expansion. Altered neuron–astrocyte interactions at striatal glutamatergic synapses, with increased striatal levels of glutamate (see [68] and references therein), seem implicated in the pathophysiology of PD; as a matter of fact, the dysfunction of astrocytes also leading to the altered control of glutamatergic transmission was proposed to have an initiating role in the PD pathophysiology [69]. Many authors have reported that both in animal models and in PD patients, the medium spiny neurons, which receive dopaminergic and glutamatergic striatal afferents, lose up to 50% of dendritic spines, and this, in rats, reflects the observed decrease in asymmetric glutamatergic synapses [70,71,72,73,74,75,76,77,78,79,80,81]. Despite this observation, there is evidence of the increased activity of the cortico-striatal system in PD, both in in vivo and in vitro models [82,83,84]. At glutamatergic axo-spinous striatal synapses in PD models, structural and neurochemical changes suggest overactivity for the cortico-striatal glutamatergic system. This is further supported by the reported increased expression of VGLUT1 in animals as well as in PD patients [85,86] and the increased density of perforated asymmetric synapses [71,87,88]. Taken together, these observations may suggest that the plastic changes in the striatal neuron–glia network in PD, and the subsequent increased release of glutamate and spillover into the extracellular space, may indirectly contribute to the loss of some spines and explain the overactive glutamatergic synapses. In this context, in the striatum, astrocytes, integral elements of the glutamatergic synapse, actively regulate the synaptic neuronal communication and plasticity, with differences in physiological and MPTP-exposed conditions, although relevant issues remain to be addressed to clearly understand the plastic changes in perisynaptic processes in the striatal neuron–glia network. In addition, dysregulated biology and the upregulation of gene expression in reactive astrocytes have been reported in PD patients [69] and associated with dopaminergic neuron degeneration. The effect of α-synuclein accumulation in astrocytes has been reported to decrease the expression of the excitatory amino acid transporters, EAAT1 and EAAT2. The best evidence for a PD-related gene with a role in astrocyte-disrupted biology is DJ-1, encoded by the *PARK7* gene [89,90]. It should be noted that in astrocytes, DJ-1 associates with lipid rafts, and regulates their assembly [91,92]. In fact, lipid rafts are highly organized microdomains at the cell membrane, and are involved in receptor trafficking, endocytosis, and signal transduction. Mutations in *PARK7* have been associated with the accelerated degradation of the lipid raft proteins flotillin-1 and caveolin-1, as well as with the induction of the disruption of lipid raft assembly [92]. Furthermore, the mutations in *PARK7* have been associated with an impaired astrocytic glutamate uptake due to a decrease in the expression of EAAT2, a glutamate transporter specifically expressed at the astrocyte membrane and previously found assembled in the lipid rafts [93]. Altogether, these observations, suggesting striatal astrocytes and glutamatergic transmission dysregulation in PD, are of particular interest in the context of the GPCR high-order complexes and on their presence and functional activity in modulating the glutamate release from striatal perisynaptic astrocyte processes. Surprisingly, although the relevance of striatal astrocyte dysfunction in pathological conditions including schizophrenia and PD, and of the striatal RRIs as possible pharmacological targets are well established, the presence and functioning of GPCR heteromers and of high-order heteromers on astrocytes, namely in striatal astrocytes, has so far hardly been investigated. By considering the relevance of receptor complexes and of neuron–astrocyte cross-talk in the signal integration, we focused our research on RRIs, in particular among GPCRs such as the D2, A2A, and OTR that might be relevant to the pathophysiology of striatal diseases, and on striatal astrocytes.

Oxytocin (OT) is a cyclic nonapeptide produced in hypothalamic neurons, magnocellular neurons located in the paraventricular and supraoptic nuclei of the hypothalamus. It is primarily released into the general circulation, acting as a hormone, from the posterior lobe of the pituitary gland (i.e., the neurohypophysis). OT-producing neurons also project to different brain areas, including the hippocampus, amygdala, striatum, hypothalamus, nucleus accumbens, locus coeruleus, the nucleus of the tractus solitarius, and the spinal cord. The release of OT could be synaptic or non-synaptic by the neuronal dendrites and spines according to wiring and volume transmission [94]. These different patterns appear to characterize the complex functions of OT as a neurotransmitter in the brain. OT has essential physiological roles in the behaviors and emotionality that promote reproduction and social interactions [95]. The oxytocin receptor OTR is found in several regions of the brain, including areas involved in the pathophysiology of neurodegenerative diseases such as Alzheimer’s disease, Parkinson’s disease, depression, anxiety, schizophrenia, autism, and attention deficit hyperactivity disorder [96]. Focusing our attention on PD, OT, and OTR have been studied in in vivo models and the treatment with the neuropeptide seems to prevent the death of dopaminergic neurons [97,98,99]. Given the cytoprotective effects of OT [100,101,102], its use in PD has been suggested [103].

In addition to the cytoprotective role that OT may have on dopaminergic neurons, oxytocinergic and dopaminergic systems seem interconnected. Their connections have been found in the process of nociception, as well as in some motivational behaviors, such as maternal and sexual behaviors, pair bonding, and salience [104]. These interlinkages are formulated based on the anatomical and functional relationship of the systems. In addition, the new type of RRI of the GPCRs for the neurotransmitter and the neuropeptide, called receptor–receptor interaction RRI, was found in the amygdala [105] as in the striatum [32,106]. In the amygdala, the dysfunction and/or disruption of the bidirectional facilitating D2-OTR interactions may lead to anxiety development. In striatal D2-OTR heteromers, OT has a facilitatory allosteric activity on the D2 receptor, promoting the receptor binding to its ligands in neurons [106]. Similarly, on astrocytes, OT facilitated the D2-mediated inhibition of the evoked glutamate release [31].

As far as striatal dopamine receptors are concerned, it is to note that in rodent PD models, the reduced function of the striatal astrocytic D2 receptor was reported to increase the vulnerability of dopaminergic neurons to MPTP, and the activation of the astrocytic D2 receptor could suppress neuroinflammation [107]. In fact, neuroinflammation seems to be crucially involved in the neurodegeneration of dopaminergic neurons [108,109,110]. In addition, our previous observations on striatal astrocytic processes revealed a functional involvement of A2A-D2 [28] and A2A-OTR heteromers [32] in the modulation of glutamate efflux. Our present finding allows us to hypothesize a functional role of a putative high-order receptor complex formed by the adenosine A2A receptor, the dopamine D2 receptor, and OTR.

Considering the A2A receptors, neuronal damage has been linked to their excessive function [111]. Interestingly, the ecto-nucleotidases responsible for adenosine formation from extracellular ATP or AMP appear abundant in striatal gliosomes in the proximity of A2A receptors [112]. Notably, A2A receptors are expressed at higher levels in the striatum in PD (see [113,114]). In PD, the increased expression of A2A receptors has been indicated as a characteristic feature of disease progression [115]. On neurons, A2A receptor activation induces an augmented efflux of glutamate, as observed from the striatal nerve terminal [28], probably linked with the increase in intracellular [Ca^2+^] and the enhancement of the long-term potentiation. All of these events may contribute to the neuronal damage through the excitotoxicity [116]. The high levels of A2A receptor expression in the basal ganglia and its functional implications in PD progression lead the A2A receptor to be considered a key pharmacological target for PD [116]. In fact, A2A receptor antagonism enhances D2 receptor-mediated signaling by the A2A-D2 RRI, and the inhibition of the A2A receptor signaling decreases motor impairment by enhancing the therapeutic effects of levodopa, and by reducing dyskinesia linked with long-term levodopa treatment [117]. On the other hand, the blockade of striatal neuronal A2A receptors could control locomotion in rodent PD models [118], while the blockade of glial A2A receptors appeared to be neuroprotective against MPTP toxicity [118]. Interestingly, the A2A receptor antagonist istradefylline is a Food and Drug Administration-approved medicine as an add-on treatment to levodopa/carbidopa in PD patients experiencing the “off” episodes [119,120,121]. In PD pharmacological current management, the chronic administration of levodopa is one of the disease’s most common therapies [122,123]. This type of pharmacological treatment is especially effective in the early stages of the disease. However, chronic administration is associated with side effects, the “off” episodes, with motor, non-motor, posture, and autonomic symptoms [123,124]. At high doses of levodopa, other impulse control disorders (compulsive spending or medication use, gambling, and abnormal sexual behaviors) have been reported. The adenosine A2A receptor overactivation is important in the pathogenesis and potentiation of “off” episodes in PD patients and they could be controlled by istradefylline, an A2A receptor antagonist [121]. In PD, istradefylline is also used to treat postural deformities [125]. The use of this drug tips the balance towards increased dopaminergic activity due to levodopa treatment, and matches the observation regarding its target expression and complex A2A-mediated function in the regulation of neurotransmission in the basal ganglia. On striatal neurons and astrocytes, A2A receptors colocalized with the D2 receptors within A2A-D2 heteromers, and with OTR in striatal astrocytes within A2A-OTR. Although the pharmacological intervention specificity would rest on selectively targeting the striatal astrocytic vs. neuronal heteromers including the A2A receptor, the blockade of A2A receptors in astrocytes might be expected to greatly impact the regulation of glutamate transmission in PD, bearing in mind the high expression of A2A receptors in PD [113,114,116], the colocalization of the striatal astrocytic A2A receptors with ectonucleotidases generating adenosine from ATP [112], and the expansion of the striatal PAPs in PD [65,66,67].

The existence of high-order molecular complexes including A2A and D2 receptors at the cell membrane has been discussed in basal ganglia neurons and co-transfected cells and includes A2A-D2-mGluR5 [126,127,128] and A2A-D2-Sigma1R heterocomplexes [129]. Considering the A2A-D2-mGluR5 heterocomplex, biochemical and behavioral evidence for multiple interactions between A2A, D2, and mGlu5R in particular in the soma-dendritic regions of the ventral striato-pallidal anti-reward GABA neurons suggested their possible implication in a heterotrimer [126,130,131]. In the A2A-D2-mGluR5 heteroreceptor complexes, A2A seems to promote the D2-mGluR5 RRI and the density of the D2-mGluR5 heteromers, while agonists for A2A and mGluR5 play a significant role in modulating the composition, density, and signaling of the A2A-D2-mGluR5 high-order complex [128]. Then, the combined use of A2A and mGluR5 agonist, targeting A2A-D2-mGluR5 heteroreceptor complexes in the ventral striatal–pallidal GABA pathway, is a new strategy for a pharmacological treatment of schizophrenia, while the use of A2A and mGluR5 antagonists may be an alternative therapeutic approach to PD [132,133]. Considering the A2A-D2-Sigma1R heterocomplex, the findings that support its existence are of high interest. Cocaine self-administration in rats increased D2-Sigma1R heteroreceptor density in the nucleus accumbens shell, while a decrease was observed in the dorsal striatum [129]. On the other hand, in the nucleus accumbens shell, but not in the core or in the dorsal striatum, cocaine self-administration increased A2A-D2 heteroreceptors without significant modifications in the density of A2A-mGlu5R heteromers in nucleus accumbens and dorsal striatum, but a reduction trend in the nucleus accumbens shell and in the dorsomedial part of dorsal striatum. In the presence of OSU-6162, a Sigma1R ligand, cocaine produced significant increases in the density of the A2A-D2 and D2-Sigma1R heteromers in the nucleus accumbens shell [134] and an A2A agonist significantly enhanced its allosteric inhibition of the D2, suggesting a cocaine-induced increased expression of Sigma1R in the ventral striatum [135]. All the results opened up the hypothesis of the existence of A2A-D2-Sigma1R heteroreceptor complexes [129,134]. In any case, the relevance of astrocytes in the pharmacological targeting of high-order receptor complexes was recently recognized by Borroto-Escuela and colleagues [136].

Here, for the first time, we provide evidence for the existence of high-order molecular complexes including A2A and D2 receptors in the striatum, namely of A2A-D2-OTR heterotrimers in the striatal astrocytes and, in astrocyte processes, for the ability of these high-order complexes to regulate the glutamatergic transmission. In fact, astrocytic D2 receptor could inhibit the evoked release of glutamate from the processes; OTR activation facilitated the D2 effect, evoking a response to a subthreshold concentration of D2 agonists, and A2A negatively controls both the D2-mediated signaling and the OTR-facilitating effect on D2. It can be suggested that the heterotrimers may represent an interesting drug target in pathological conditions involving the dysregulation of striatal astrocytic function and of glutamatergic transmission. In fact, the astrocytic receptor complex might allow one to design novel therapeutic approaches to counteract a defective activation of striatal astrocytic D2 receptors in PD, responsible for increased and dysregulated glutamate release (potentially leading to excitotoxicity) and impaired neuroprotection against injury-induced neuroinflammation, neurodegeneration, and synaptic dysfunction [109,110]. In PD, indeed, OTR may be targeted to facilitate dopaminergic transmission onto astrocytic D2, making the D2 receptor responsive to subthreshold dopamine concentrations, while the blockade of the inhibitory control exerted by A2A onto both D2 and OTR may further enhance the astrocytic D2 receptor functioning. The facilitation of the astrocytic D2 receptor functioning may lead to a reduction in glutamate release [28,32] and therefore, of the potential excitotoxicity but also to a reduction in neuroinflammation (see [107]) which might be relevant to PD treatment. Indeed, the activation of the OTR and blockade of the A2A receptor in the receptor complex in PD might contribute to the rescue of the control of glutamate extracellular levels and the impaired anti-neuroinflammatory effect resulting from the defective activation of astrocytic D2 receptors. It is to be noted that this novel approach can be only proposed based on the evidence for the existence (and functioning) of the receptor complex, which allows one to design selective intervention on targeting supramolecular structures to selectively target cell types and functions that can be involved in the disease pathogenesis.

A2A-D2-OTR complex was investigated here on striatal astrocytes, but we cannot exclude that the mosaic could be formed on neurons or other glial cells.

In conclusion, the D2 receptor appears to be a hotspot in the control of glutamate release from the astrocytic processes and may contribute to the regulation and integration of different neurotransmitter-mediated signaling in the striatum. GPCR high-order receptor complexes may offer selective pharmacological targets, due to the possible selectivity of allosteric interventions on GPCRs organized as receptor mosaics at specific neuron–astrocyte networks, and a way to increase the selectivity of pharmacological treatments. The A2A, D2, OTR, and the mosaicism in which they are involved in astrocytes may be potential therapeutic targets in neuropsychiatric disorders and neurodegenerative diseases. Notably, the ability of the allosteric modulators of the D2 receptor (proposed as promising therapeutic approaches to neuropsychiatric/neurodegenerative diseases including schizophrenia or PD [137]); to impact the D2 receptor involvement in mosaics would also deserve investigation. Furthermore, the molecular and intracellular pharmacology of OTR, as a single player as well as in heteromers or high-order receptor complexes, remains to be elucidated. The molecular cascade of events, the knowledge of which will significantly advance our understanding of the OT effects on the brain and its role in different disorders, necessitates further research.

## 4. Materials and Methods

### 4.1. Animals

Sprague–Dawley rats (adult males weighing approximately 200–250 g) were housed in the animal facility of the Department of Pharmacy (DIFAR), University of Genova, Genova, Italy. The housing conditions were constantly controlled (22 ± 1 °C; 50% relative humidity; 12 h light–dark cycle, with light from 7 am to 7 pm), and the animals received a standard diet and water ad libitum. Animal care was performed in agreement with the European Communities Parliament and Council Directive 2010/63/EU and with the Italian D.L. n° 26/2014. The experimental procedures were approved by the Italian Ministry of Health (protocol number 30/11/2016-OPBA of November 2016), in accordance with Decreto Ministeriale 116/1992. Every effort was made to minimize suffering and to reduce the number of rats used.

### 4.2. Slices Preparation and Cresyl Violet Staining

As described previously [31,32], the whole brain was removed immediately after decapitation. To prepare cryostat sections, the brain was divided in the right or left half and they were embedded in paraformaldehyde 4% in PBS (24 h at 4 °C). The day after, the tissue was transferred in sucrose 30% in PBS (24 h at 4 °C) and then into the inclusion matrix to prepare the tissue sample for cryostat cutting, Killik O.C.T. (Bio-Optica, Milan, Italy). The tissue was finally frozen in liquid nitrogen and conserved at −20 °C until the preparation of coronal 10–15 µm thick slices with a cryostat (Leica CM1900UV, Wetzlar, Germany). The slices were placed on poly (L)-lysine precoated slides and stored at −20 °C until further processing. The selected sections were thawed at RT, stained with cresyl violet (Bio-Optica), differentiated in two changes of 95% ethanol, rapidly dehydrated in absolute ethanol, cleared with Bio-clear (Bio-Optica), and permanently mounted with Eukitt (Bio-Optica). Whole slide images were acquired using the Manual WSI software 2020C-34FL (Microvisioneer, Esslingen am Neckar, Germany) and a BX60 microscope (Olympus, Hamburg, Germany) equipped with a color camera (Basler, Ahrensburg, Germany).

### 4.3. Proximity Ligation Assay (PLA) and Immunofluorescent Confocal Microscopy on Slices

To assay the presence of the A2A, OTR, and D2 receptors on mature striatal astrocytes, we carried out an immunofluorescence assay with a classic double immunostaining. The rat hemibrain slices were permeabilized and treated with Blocking Solution, then incubated in a humid chamber over-night (O.N.) at 4 °C with the following primary antibodies: mouse anti-A2A receptor (1:200; Merck Millipore Corporation, Milan, Italy), rabbit anti-D2 receptor (Alomone Labs, Jerusalem, Israel), rabbit anti-OTR (Alomone Labs), and goat anti-GFAP antibodies (1:500, Santa Cruz Biotechnology Inc., Dallas, TX, USA). To validate the goat anti-GFAP antibody, we performed a double immunostaining using also mouse anti-GFAP antibody (1:5000, Sigma-Aldrich, Milan, Italy; Figure 1K–M). The day after, the slices were incubated with the opportune secondary antibodies Alexa Fluor 488, 546, or 633-conjugated (from 1:500 to 1:1000; Life Technologies Corporation Corporation, Carlsbad, CA, USA) or Atto 647-conjugated (1:1000; Sigma-Aldrich) and diluted in PBS containing 3% BSA. After washing in PBS, the slides were overlapped with ProLong Gold mounting medium (Thermos Fischer Scientific, Milan, Italy) and a coverslip. To seal the edges of coverslips to slides, we used nail polish. The slides were stored at 4 °C until confocal image acquisition.

To assess the colocalization of the A2A, OTR, and D2 receptors, and, in particular, of A2A with the D2-OTR heteromers, we carried out the in situ PLA between the OTR and D2 receptors on the rat hemibrain slices and the immunolabeling of the A2A receptors. Firstly, the PLA was carried out as previously described [30,31,32] by pre-conjugating the primary rabbit anti-D2 receptor (Alomone Labs) and rabbit anti-OTR (Alomone Labs) antibodies with the PLA PLUS and PLA MINUS oligonucleotides, respectively, using the Duolink in situ Probemaker kit (PLUS DUO92009, MINUS DUO92010, Sigma-Aldrich) following the manufacturer’s instructions. PLA OTR-D2 was performed using the Duolink Detection Kit (DUO92014, DUO92002, DUO92004 Sigma-Aldrich) as indicated in the kit instructions. The slices, previously permeabilized and treated with Blocking Solution, were incubated with conjugated anti-D2 PLUS and anti-OTR MINUS antibodies in a humid chamber over-night (O.N.) at 4 °C. The hybridization, ligation, and amplification steps were performed, as manufacturer’s instruction, to allow the visualization of the D2-OTR heteromers by green fluorescence. In parallel, in the negative control slices, one of the conjugated primary antibodies was omitted, resulting in a complete lack of PLA stain [32]. To co-localize the heteromers with the A2A receptors on astrocytes, the slices were incubated (humid chamber, O.N., 4 °C) with the mouse anti-A2A receptor, and then with the Alexa Fluor 546-conjugated donkey anti-mouse (1:500; Molecular Probes, Eugene, OR, USA; 1 h at room temperature, RT). To avoid the cross-reaction, a sequel protocol was applied. After a new saturation with BSA 0.5% in PBS, the slices were incubated with the goat anti-GFAP (1:500, Santa Cruz Biotechnology Inc.) and mouse anti-ezrin (1:100; Sigma-Aldrich) antibodies. The used secondary antibodies were incubated for 1 h at room temperature, RT. We used Alexa Fluor 633-conjugated donkey anti-goat (1:1000; Life Technologies Corporation) and Atto 647-conjugated anti-mouse (1:1000; Sigma-Aldrich). The combination of fluorochromes was chosen to avoid crosstalk. The slides were closed with coverslips using mounting medium (DUO82940) and nail polish, and then stored at 4 °C until the confocal analysis.

The labeled slices were analyzed by Leica STELLARIS 8 Falcon τSTED (Leica Microsystems, Mannheim, Germany) inverted confocal/STED microscopy. A white light laser provided the excitation that was acquired by three Power HyD detectors. An HC PL APO CS oil immersion objective 100× (1.40 NA) was used to acquire the fluorescence images (1024 × 1024 × 16 bit) with a line scanning speed range at 400 Hz and setting the pinhole at 1 Airy size. The Leica “LAS X application Suite” software package 4.4.0.24861 was used for acquisition, storage, visualization, and 3D analysis.

To display a two-dimensional graph of the gray intensities of pixels along a line, we used the profile plot tool in the ImageJ Fiji software 2.9. We drew a yellow line (Figure 2J) and applied it to all the channels. We generated three plots and the merge plot (Figure 2K).

### 4.4. Preparation of Purified Striatal Astrocytic Processes

The striatum was dissected and placed in an ice-cold solution (0.32 M sucrose, 10 mM Tris/HCl; pH 7.4). The purified astrocyte processes (gliosomes) were prepared according to Nakamura’s protocol [138]. The tissue was homogenized in a sucrose solution and the homogenate was rapidly centrifugated to remove debris and nuclei. The supernatant was gently stratified on a discontinuous Percoll gradient (2, 6, 10, and 20% (*v*/*v*) in Tris-buffered sucrose) and centrifugated to collect the gliosomes at the interface between 2 and 6% Percoll solution. A final centrifugation ensured the complete removal of Percoll and allowed us to transfer the gliosomes in the final HEPES standard medium used for the other experimental procedures. The used HEPES medium (pH 7.4) had the following composition (mM): NaCl 128, KCl 2.4, MgSO_4_ 1.2, KH_2_PO_4_ 1.2, CaCl_2_ 1.0, and HEPES 10 with glucose 10. Gliosomes were previously shown as a purified preparation of astrocytic processes. With the immunofluorescent assay and Western blot analysis, we demonstrated that they are negligibly contaminated by neuronal subcellular particles [29,139,140]. Previously, we observed that striatal gliosomes were positively labeled for GFAP and for ezrin, astrocytic marker, and selective marker restricted to the perisynaptic processes, respectively [141]. Gliosomes were positively labeled with the VGLUT1, and released endogenous glutamate when stimulated with 4-AP in a Ca^2+^-dependent manner [28]. The activation of the A2A receptors proved ineffective in inducing a glutamate-releasing response while the same stimulus increased the glutamate outflow in striatal synaptosomes, nerve terminals that are obtained in parallel with the striatal gliosomes [28].

### 4.5. Immunofluorescent Labeling in Gliosomes and Confocal Microscopy

To assess by immunofluorescence confocal analysis the presence of the studied GPCRs on striatal astrocyte processes, gliosomal preparations were used as previously described [28,31,32]. The gliosomes (1 μg/μL in standard HEPES medium) were treated with paraformaldehyde 2% in phosphate-buffer saline (PBS). Using 0.05% Triton X-100 in PBS plus 3% bovine serum albumin (BSA), the gliosomes were permeabilized and saturated. The primary antibodies were diluted in PBS supplemented with 3% BSA and then incubated (O.N., 4 °C in a humid chamber) with the gliosomes. To avoid the cross-reaction, a sequel protocol was applied. The primary antibodies used were goat anti-GFAP (1:500; Santa Cruz Biotechnology Inc.), rabbit anti-D2 receptor (1:200; Alomone Labs), rabbit anti-OTR (1:200; Alomone Labs), and mouse anti-A2A receptor (1:200; Merck Millipore Corporation). The day after, the gliosomes were incubated with a secondary antibody Alexa Fluor 488, 546, or 633-conjugated (1:1000; Life Technologies Corporation) and Atto 647-conjugated (1:1000; Sigma-Aldrich) and diluted in PBS containing 3% BSA. After washing in PBS, the labeled gliosomes were placed on slides, overlapped with the ProLong Gold mounting medium (Thermos Fischer Scientific) and a coverslip. To seal the edges of coverslips to slides, we used nail polish. As for the slices, the slides were stored at 4 °C until the confocal image acquisition. For each condition, a negative control sample was prepared omitting only a primary antibody and obtaining the complete lack of the relative stain. The slides were analyzed by the Leica STELLARIS 8 Falcon τSTED inverted confocal/STED microscope as described above for the slices.

### 4.6. Immunoprecipitation and Immunoblotting Experiments

The freshly prepared gliosomes (1 mg/mL) were resuspended in a lysis buffer with the follow composition: 50 mM sodium borate buffer (pH 7.5), 1 mM EDTA, and Protease Inhibitor Cocktail. The gliosomes were lysed by sonication and we performed the protein quantification of the gliosomal lysate according to the Bradford method [142]. After that, the lysate was centrifuged at 100,000× *g* for 30 min at 4 °C, the pellet was washed and solubilized (37 °C for 1 h) in 100 µL immunoprecipitation buffer with the following composition: 50 mM sodium borate, 0.1 mM EDTA, and pH 7.5 + 1% Triton-X100. The Triton-X100 was diluted to 0.2% with 400 µL of immunoprecipitation buffer. Then, the gliosomal lysate was centrifuged at 18,000× *g* for 15 min at 4 °C and the supernatant was precleared with protein G sepharose™, and then incubated over-night in the presence of 1 µg of anti-OTR, or anti-A2A receptor, or anti-D2 receptor antibody at 4 °C. Protein G sepharose™ was then added to the samples and incubated for an additional 1 h at room temperature (RT). The immunocomplexes were washed three times with immunoprecipitation buffer + 0.1% Triton-X100, heated at 95 °C in SDS-PAGE loading buffer for 5 min, and submitted to 10% SDS-PAGE followed by Western blot on 0.45 µm nitrocellulose membrane. The membranes were probed with specific antibodies and the immunoreactive material was detected using the ECL select™ Western blotting detection reagent and Chemi Doc XRS apparatus (Bio-Rad Laboratories, Segrate, Italy). To test each blot for the four antigens, the membranes were submitted to a stripping and re-probing procedure (see ECL select™ manufacturer’s instructions for details).

### 4.7. Endogenous Glutamate Release

The release of the endogenous glutamate was studied superfusing a gliosomal monolayer with a standard medium maintained at 37 °C. Striatal astrocyte processes were stratified at the bottom of the superfusion chambers, and the superfusion rate was 0.5 mL/min. This up–bottom superfusion technique avoids the generation of a receptor biophase and any indirect effect that the released substances might have on neighboring gliosomes [28,31,32,143]. The HEPES medium could be supplemented with any pharmacological tool (agonists and antagonists) according to experimental design. In this way, it is possible to characterize the pharmacological profile of a receptor and explore the interactions that could occur among pharmacological targets [144,145]. The release experiment starts with a superfusion period (33 min) in which gliosomes stabilize the basal glutamate outflow. From t = 33 min, for each chamber five superfusion fractions were collected as 3 min samples (B1–B5), and the first two fractions were used to evaluate the basal outflow of glutamate. At t = 38 min, the gliosomes were stimulated with the depolarizing stimulus (300 µM 4-AP; 6 min) alone or with the A2A or D2 agonists and/or OT. The selective drugs and their concentrations used for the pharmacological characterization of the studied receptors are reported in Table 1.

When we evaluated the A2A antagonist effect, SCH-58261 was added 8 min before the agonists. For control, at least one chamber was superfused only with the standard medium or with a medium appropriately modified (e.g., added with antagonists) in each experiment. As previously observed, OT 3 nM was effective in inhibiting the glutamate release evoked by 4-AP through OTR activation [31,32], as well as in facilitating the D2-mediated response in glial OTR-D2 heteromers [32]. CGS 21680 0.01 µM was effective in enhancing the glutamate release from purified striatal nerve terminals [28] and in modulating the D2 receptor control or the OTR mediate inhibition of the 4-AP-evoked glutamate efflux from the striatal gliosomes [28,29,31,32]. Quinpirole 0.1 µM was the ineffective concentration in the modulation of the 4-AP-evoked glutamate release, but it was used to assess the D2-OTR heteromers in purified striatal PAPS [32].

The glutamate released in the collected samples was expressed as pmol/mg protein. The protein determination was carried out on each gliosomal preparation according to Bradford’s method [142]. To measure the glutamate, an HPLC analysis was carried out as previously described [146]. The method involved the automatic precolumn derivatization of the amino acids with O-phthalaldehyde; the derivatization products were separated on a C18 column and detected by a fluorometric detector. The method required homoserine as an internal standard and a standard solution with a known concentration of glutamate for the calibration curve. For each chamber, the mean of glutamate amounts released in B1 and B2 (the two basal fractions) was taken as the 100% control value. The amounts of glutamate released in the other fractions (B3–B5) were then evaluated as the percent variations with respect to the control value. The chambers superfused only with the standard medium were considered as control conditions. The chambers superfused with the standard medium added with an antagonist were considered as the control condition for all the chambers in which the antagonist was present. To evaluate the effect of an agonist, the drug effect was measured by subtracting the percent variations in glutamate efflux in the control condition from the percent variations in the drug-presence conditions.

### 4.8. Intracellular [Ca^2+^] Assay

Cytosolic gliosomal [Ca^2+^]_i_ was determined as previously described [31,147]. Briefly, the freshly prepared gliosomes were washed once in HEPES buffer and then incubated with 10 μM Calcium Green™-1 AM (CG; 30 min at 37 °C) in the same buffer. After two washes in HEPES buffer, the gliosomes were transferred to black 96-well plates (50 μg/well) and exposed to drugs (see Table 1). Using the top reading mode of the LB940 Mithras Fluorescence Multi-Label Reader (Berthold Technologies, Baden Württemberg, Germany), fluorescence intensities (excitation 485 nm, emission 535 nm) were measured every 10 s for 5 min. For each recording, the fluorescence value measured at the start time was subtracted. At each recording time, the [Ca^2+^]_i_ variation (Delta Fluorescence) was calculated as the difference between the CG-dependent fluorescence of the stimulated samples and that of the vehicle-treated samples. The time courses of CG-dependent [Ca^2+^]_i_ variations obtained in the vehicle-treated and stimulated samples were used to calculate the area under the curve (AUC) for each experimental condition.

### 4.9. Modeling of the A2A-D2-OTR Mosaic

The experimentally assessed molecular structures of human adenosine A2A, dopamine D2, and oxytocin receptor (OTR) were retrieved from the Protein Data Bank [148]. A2A (PDB code: 3PWH [149]), D2 (PDB code: 6CM4 [150], and OTR (PDB code: 6TPK [151] were experimentally obtained by X-ray diffraction at a resolution of 3.30 Å, 2.87 Å, and 3.20 Å, respectively.

By using the DockPrep module available in the UCSF Chimera molecular modeling software (rel. 1.17.1, Resource for Biocomputing, Visualization, and Informatics, University of California, San Francisco), all extra molecules (such as ligands) were removed, hydrogens were added, and partial charge assigned. The obtained molecular structures were then energy minimized by using the Yasara software [152,153] and stored for further processing.

The structures of the possible heterotrimers of A2A, D2, and OTR were estimated by using the Mem-LZerD software [154] allowing the docking of the structures under the assumption that they are integral with the same membrane [155]. Since this software can manage only two protein structures, a two-step procedure was performed. In the first step, the three receptors were docked in pairs and for each pair, the solution exhibiting the best energy score was selected as the heterodimeric structure. In the second step, the docking between each selected heterodimer and the third receptor was analyzed. The obtained solutions exhibiting the correct orientation of the protomers were then ranked by energy score and for each estimated topological arrangement (defined in terms of the number of interfaces per protomer), the best solution was considered as a possible heterotrimeric structure.

To refine the selected solutions using a more realistic model of the biological environment, each structure was inserted into a pre-equilibrated 1-palmitoyl-2-oleoylphosphatidylcholine (POPC) bilayer (see Figure 7A) using CHARMM-GUI Membrane Builder [156,157]. Energy minimization was then performed by using the Yasara software http://www.yasara.org/minimizationserver.htm (accessed on 13 March 2024) [152,153].

The structural features of the predicted heteromerization interfaces were explored by the PDBePISA tool [158] freely available [159].

### 4.10. Calculations and Statistical Analysis

The means ± SEM of the numbers of experiments (n) are indicated throughout. Data distribution was evaluated by means of the Kolmogorov–Smirnov normality test. The significance of the difference as well as the determination of the AUC was analyzed by the non-parametric Mann–Whitney test (if data were not normally distributed or *n* < 5) or by the parametric one-way ANOVA and Tukey’s post hoc test, with statistical significance being taken at *p* < 0.05. Statistical analysis was carried out using the Prism 4.02 software package (GraphPad Software, San Diego, CA, USA).

### 4.11. Materials

4-Aminopyridine (4-AP), Triton-X 100, CGS 21680, SCH-58261, quinpirole, and oxytocin (OT) were purchased from Sigma-Aldrich. The drugs were dissolved in distilled water. All the salts were from Sigma-Aldrich or from VWR. Calcium Green™-1 AM was from Life Technologies Italia (Milan, Italy). Protease Inhibitor Cocktail (100X), horseradish peroxidase (HRP)-linked anti-mouse, and anti-rabbit secondary antibodies from Cell Signaling Technology; Amersham™ ECL select™ Western blotting detection reagent and Amersham™ Protran™ 0.45 µm nitrocellulose membrane from Cytiva; prestained protein SHARPMASS VI MW markers; and protein G sepharose™ from GE Healthcare were all purchased from Euroclone (Milan, Italy). The primary antibodies (rabbit anti-OTR, rabbit anti-A2A receptor, and rabbit anti-D2 receptor) were purchased from Alamone Labs (Jerusalem, Israel), while mouse anti-flotillin-1 from BD Biosciences was purchased from DBA Italia (Segrate, Italy).

## Figures and Tables

**Table 1:** Pharmacological tools and their concentration used to modulate the endogenous glutamate efflux or the cytosolic [Ca^2+^].

	A2A	D2	OTR
Agonist	CGS 21680	quinpirole	oxytocin (OT)
concentration	0.01 µM	0.1 µM	3 nM

## Data Availability

Data is available upon request from the corresponding authors.
